# 2021 FDA TIDES (Peptides and Oligonucleotides) Harvest

**DOI:** 10.3390/ph15020222

**Published:** 2022-02-13

**Authors:** Danah Al Shaer, Othman Al Musaimi, Fernando Albericio, Beatriz G. de la Torre

**Affiliations:** 1 KRISP, School of Laboratory of Medicine and Medical Science, College of Health Sciences, University of KwaZulu-Natal, Durban 4001, South Africa; danah.shaer@gmail.com (D.A.S.); garciadelatorreb@ukzn.ac.za (B.G.d.l.T.); 2 Surfaces and Particle Engineering Laboratory, Department of Chemical Engineering, Imperial College London, London SW7 2AZ, UK; musamiau@gmail.com; 3 School of Chemistry and Physics, University of KwaZulu-Natal, Durban 4001, South Africa; 4 CIBER-BBN, Networking Centre on Bioengineering, Biomaterials and Nanomedicine, Department of Organic Chemistry, University of Barcelona, 08028 Barcelona, Spain; 5 Institute for Advanced Chemistry of Catalonia (IQAC-CSIC), 08034 Barcelona, Spain

**Keywords:** drugs, FDA, oligonucleotides, peptides, antibody-drug conjugate, inclisiran, casimersen, vosoritide, melphalan flufenamide, voclosporin, pegcetacoplan, dasiglucagon, Piflufolastat-F^18^, difelikefalin, odevixibat, tisotumab vedotin-tftv, loncastuximab tesirine-lpyl

## Abstract

From the medical, pharmaceutical, and social perspectives, 2021 has been a year dominated by the COVID-19 pandemic. However, despite this global health crisis, the pharmaceutical industry has continued its endeavors, and 2021 could be considered an excellent year in terms of the drugs accepted by the US Food and Drug Administration (FDA). Thus, during this year, the FDA has approved 50 novel drugs, of which 36 are new chemical entities and 14 biologics. It has also authorized 10 TIDES (8 peptides, 2 oligonucleotides), in addition to 2 antibody-drug conjugates (ADCs) whose structures contain peptides. Thus, TIDES have accounted for about 24% of the approvals in the various drug categories. Importantly, this percentage has surpassed the figure in 2020 (10%), thus reflecting the remarkable success of TIDES. In this review, the approved TIDE-based drugs are analyzed on the basis of their chemical structure, medical target, mode of action, administration route, and adverse effects.

## 1. Introduction

Like the previous year, 2021 has been dominated by the COVID-19 pandemic from the medical/pharmaceutical and social perspectives. At the end of 2020, public health analysts predicted that massive vaccination campaigns would bring about a new kind of “normal” to daily life. This has been achieved in part. The vaccination program has greatly reduced morbidity and mortality from the disease, but the appearance and massive spread of the omicron variant towards the end of 2021 have led to a feeling of social insecurity worldwide. However, the variants driving the pandemic have not impeded the pharmaceutical industry from having an excellent year in terms of new approvals by the US Food and Drug Administration (FDA). In this regard, in 2021 (almost referred to herein as “this year”), the FDA has approved 50 drugs, thus bringing the total number of authorizations to 278 over the last six years [1]. A total of eight peptides, two oligonucleotides, and two ADCs with peptide fragments have been approved this year. These figures reflect the best TIDES harvest to date.

**Figure 1:**
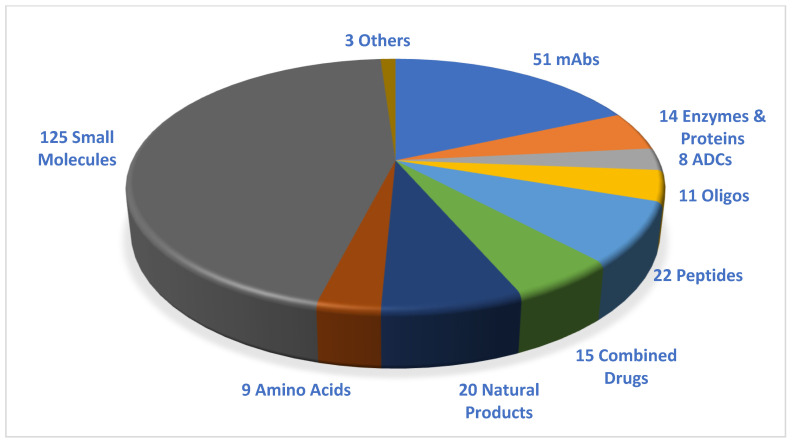
A total of **278** new drugs were approved by the Food and Drug Administration (FDA) from 2016 to 2021. Reprinted from ref. [1]. mAbs, monoclonal antibodies; ADCs, antibody-drug conjugates; Oligos, oligonucleotides.

Considering the drugs accepted in the period 2016–2021 (Figure 1), TIDES account for almost 13% (35 vs. 278) of the total number of drugs approved [2].

The peptide drugs approved have a variety of structures, including linear, homodetic cyclic, disulfide bridge, and peptide mimics (urea from two amino acids). Furthermore, peptides are present as payloads and as linkers in ADCs. Interestingly, two of the peptides approved, namely voclosporin (Lupkynis™) and odevixibat (Bylvay^TM^), are administered orally, while studies into the oral administration of difelikefalin (Korsuva^TM^) are also underway. The delivery of orally available peptides has always been a concern and a challenge. Having said that, two other peptides, plecanatide (Trulance™) and macimorelin (Macrilen™), which are also administered orally, were approved in 2017 [3]. Thus, with Lupkynis™ and Korsuva^TM^, a total of four orally available peptides have received authorization in the last five years.

The TIDES approved in 2021 are summarized in Table 1, along with their indication, therapeutic target, and administration route.

## 2. Oligonucleotides

Two oligonucleotide drugs were approved in 2021, a double-strand short interfering RNA (siRNA) and an antisense polymorpholino-oligomer (PMO)-based oligonucleotide, mimicking those two approved in 2020, also belonging to the same class, namely Lumasiran (Oxlumo^TM^) and Viltolarsen (Viltepso^TM^), respectively. Since 1998, when the first oligonucleotide (Fomivirsen, Vitravene^®^) was approved by the FDA, 13 more have been authorized, all this century. Eleven of these have reached the market since 2016. Oligonucleotides have needed approximately 20 years for their consolidation as drugs. This exemplifies the difficulty faced by the pharmaceutical industry.

### 2.1. Inclisiran (Leqvio^TM^)

**Figure 2:**
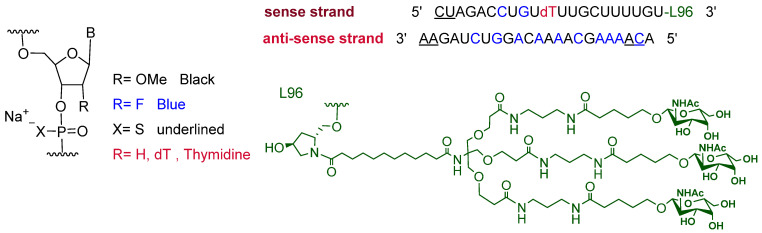
Chemical structure of inclisiran (Leqvio^TM^).

It is a siRNA. The sense strand is composed of twenty-one monomers, one of which holds thymidine, which introduces three chiral centers to the strand, unlike other units that introduce four each. The strand has two phosphorothioate linkages at its 5′ end, and it is attached to the hydroxy proline-N-acetylgalactosamine (GalNAc) dendrimer, L96, through its 3′ terminus. The antisense strand comprises 23 complementary sequences with 2 phosphorothioate linkages at each end (Figure 2) [4]. Inclisiran is the third drug in which enhanced stabilization chemistry (ESC) within the double strands is conjugated to GalNAc ligand, as previously used in Lumasiran [5] and Givosiran [6]. The latter two drugs were approved by the FDA in 2020 and 2019, respectively. All three drugs are driven by L96, which targets asialoglycoprotein receptors (ASGPR) on hepatic cells [7].

Inclisiran is the first-in-class of siRNA drugs for the treatment of hypercholesterolemia (heterozygous familial and nonfamilial) or mixed dyslipidemia, alone or in combination with other lipid-lowering therapies [7,8], such as statins. The latter are small molecule drugs commonly used to bring down low-density lipoprotein (LDL) levels in patients. In some cases, these drugs may have limited efficiency in lowering LDL levels even when the maximum tolerated dose is administered. Their efficiency can be boosted further by using inclisiran [7]. However, inclisiran can be administered with or without other LDL-reducing drugs [4].

Hypercholesterolemia is a condition that results from elevated levels of some circulating lipoproteins (all except the high-density lipoprotein HDL-cholesterol), namely low-density and medium-density lipoproteins in the bloodstream. These lipoproteins carry insoluble cholesterol in blood plasma, and elevated levels of them can cause atherosclerosis and coronary heart disease [9]. This increase in lipoproteins may result from unhealthy diets, obesity, type II diabetes, underactive thyroid, or genetic mutations that affect LDL receptors. Some of these mutations affect the proprotein convertase subtilisin/kexin type 9 (PCSK9) (a protein responsible for the expression and recycling of LDL protein receptor to the cell surface), thus decreasing LDL uptake by the cell and consequently increasing its level in the bloodstream [4,7]. After entering the hepatic cell, inclisiran targets and prevents the translation of PCSK mRNA, thus blocking the production of this protein, preventing the degradation of LDL receptors, and, as a result, lowering LDL levels in blood [4,7,8].

Inclisiran is administered subcutaneously, and it is well tolerated. However, it has some adverse effects at the injection site, such as pain, rash, or erythema [7,8,9]. It was developed, along with other PCSK9-targeting therapeutics, by Alnylam Pharmaceuticals Inc. (Cambridge, MA, USA) until it reached phase I clinical trials. Then, under a collaboration agreement, Novartis (East Hanover, NJ, USA) proceeded with phase II clinical trials onwards [7] until its final approval in December 2020 in Europe. It received FDA approval later, on 22 December 2021, in the US [10] due to delays caused by the global pandemic.

### 2.2. Casimersen (Amondys 45)

**Figure 3:**
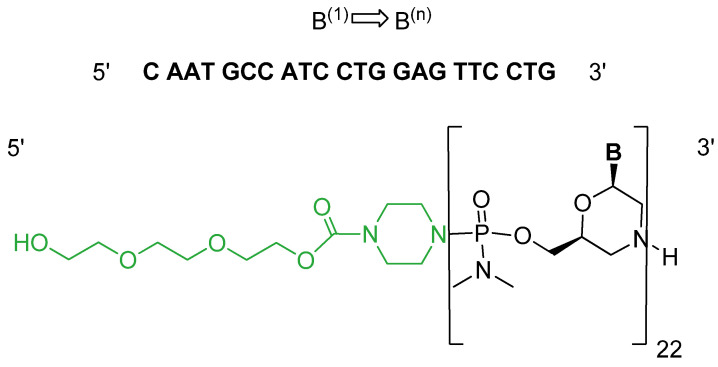
Chemical structure of casimersen (Amondys 45).

It is an antisense single-strand polymorpholino-oligomer (PMO) with phosphodiamidate linkages. Casimersen consists of 22 bases linked to a triethyleneglycol (miniPEG) tail at the 5′ end (Figure 3) [11].

Casimersen is the fourth antisense oligonucleotide drug that uses an exon-skipping mode of action for the treatment of Duchenne muscular dystrophy (DMD). DMD is a genetic disorder in which the transcription of the Dystrophin gene and consequently the translation of the dystrophin protein is interrupted due to deletion mutations, which result in the production of non-functional dystrophin. Dystrophin is a protein that covers and protects muscle fibers against deterioration upon contraction, and its deficiency causes muscle wasting and eventually death, mainly by a heart attack. Some mutations are amenable for exon skipping and consequently allow the retarded transcription reading frame to proceed and the dystrophin protein, which is shorter than normal, to be produced [2,12]. To date, three exon-skipping drugs have reached the market. Casimersen is used to treat DMD cases caused by mutations that are responsive to exon 45 skipping [11], while golodersin [13] and viltolarsen [14] are used for exon 53 and eteplirsen [15] for exon 51 skipping.

Casimersen is administered intravenously, and the most common adverse effects are upper respiratory tract infection, cough, pyrexia, headache, arthralgia, and oropharyngeal pain [11]. Developed by Sarepta Therapeutics, Inc. (Cambridg, MA. USA), it was granted accelerated approval by the FDA on 25 February 2021 [16].

## 3. Peptides

If the two ADCs are taken into consideration, 2021, with ten peptide approvals, has been the best year on record for peptides authorized by the FDA, even surpassing 2017, when six peptides were given the green light. From a structural perspective, the peptides authorized in 2021 represent a mini-course on peptide chemistry. The approvals include large, medium, and small peptides, cyclic through both lactam or disulfide bonds, pegylated peptides, and mimetic peptides where two amino acids are bound through a carbonyl group, forming urea. From a synthetic perspective, some of these peptides are produced by solid-phase synthesis, while others are prepared by recombinant technology, semi-synthesis, or solution chemistry.

### 3.1. Vosoritide (Voxzogo^TM^)

**Figure 4:**
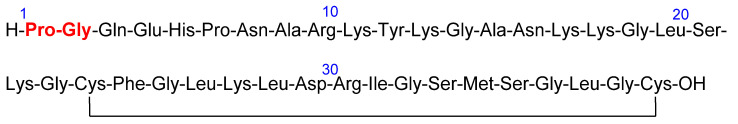
Chemical structure of vosoritide (Voxzogo^TM^). The extra Pro-Gly with respect to human CNP53 is shown in red.

Vosoritide is a 39 amino acid peptide that belongs to a C-type natriuretic peptide (CNP). It is an analog of CNP53, and it contains the 37 *C*-terminal amino acid residues of human CNP53, in addition to Pro-Gly (red) at the *N*-terminus to tackle neutral endopeptidase (NEP) degradation, thus increasing its half-life [17]. The presence of the disulfide bridge between the two Cys residues also serves the same purpose [17] (Figure 4).

Vosoritide is prescribed for the treatment of a genetic disorder called “achondroplasia” [17,18], the most common form of disproportionate short stature. This condition is caused mainly by a gain-of-function pathogen variant in the fibroblast growth factor receptor 3 gene (FGFR3) [17]. FGFR3 regulates bone growth, and its overactivation inhibits endochondral ossification [19]. Vosoritide was designed to mimic CNP. Thus, it binds to natriuretic peptide receptor B (NPR-B), which subsequently stimulates intracellular cyclic guanosine monophosphate production (cGMP) and inhibits the effect of FGFR3, and promotes endochondral bone growth by stimulating chondrocyte proliferation and differentiation [20]. Interestingly, vosoritide is the first treatment of achondroplasia with a precise therapy, whereas the other treatments available focus on the management of symptoms [19]. Of note, another C-type natriuretic drug called TransCon CNP, engineered to be administered weekly for the treatment of achondroplasia and with a longer half-life than vosoritide, is currently in the clinical development stage [21].

Vosoritide is administered subcutaneously. Various adverse effects are associated with it, including injection site erythema, swelling and urticaria, vomiting, decrease in blood pressure, and gastroenteritis [18]. It was developed by BioMarin Pharmaceutical (Novato, CA, USA) and is manufactured from *Escherichia coli* (*E. coli)* via recombinant DNA technology [18]. In March 2016, Chugai Pharmaceutical (Chuo City, Tokyo, Japan) finalized an exclusive sublicense agreement with BioMarin Pharmaceutical (Novato, CA, USA) on the patent of Chugai Pharmaceutical’s CNP (Chuo City, Tokyo, Japan) to make vosoritide available for patients. This drug was approved in the EU on 27 August 2021 [17] and then by the FDA on 19 November of the same year [22].

### 3.2. Melphalan Flufenamide (Pepaxto^®^)

**Figure 5:**
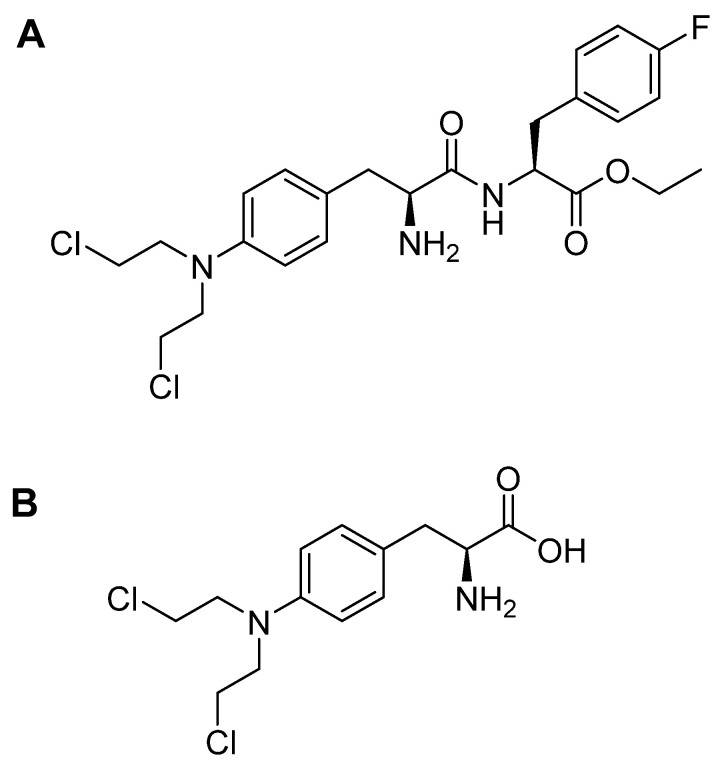
Chemical structures of: **A**. melphalan flufenamide (Pepaxto^®^); **B**. melphalan.

Melphalan flufenamide is an ethyl ester lipophilic peptide-inspired amide-containing drug that consists of melphalan and p-fluoro-L-phenylalanine (Figure 5A). The high lipophilicity of melphalan flufenamide facilitates its cellular uptake, which is followed by its hydrolysis with the aid of peptidases. Given the simple amide bond in this drug, it can be degraded mainly by aminopeptidases such as aminopeptidase N (also called CD13), which is overexpressed in various tumor cells. This degradation eventually leads to the release of the melphalan metabolite (Figure 5B) and its transport across the cell membrane by passive diffusion [23]. A prodrug of melphalan, melphalan flufenamide, exerts anti-tumor activity through crosslinking of DNA [23].

Melphalan flufenamide is prescribed for multiple myeloma (MM) and amyloid light-chain amyloidosis, and usually in combination with dexamethasone [23,24]. It also shows anti-tumor activity against various tumors (MM, lymphoma, and acute myeloid leukemia cell lines, among others) [23]. It can also inhibit MM cell migration and tumor-associated angiogenesis [25]. Most importantly, it can induce cell apoptosis even in melphalan- and bortezomib-resistant MM cells [23,25], especially when administered with dexamethasone, and this is known as synergistic cytotoxic activity [23]. Ray and co-workers suggested that the capacity of melphalan flufenamide to overcome such resistance is attributable to its ability to trigger rapid and irreversible DNA damage. In contrast, free melphalan upregulates Ku80, which repairs DNA double-strand breaks [26]. A study by Byrgazov and co-workers concluded that melphalan flufenamide could be considered an adjuvant to doxorubicin, improving therapeutic efficacy for the treatment of metastatic high-grade osteosarcoma (HGOS) [27]. Interestingly, low doses of melphalan flufenamide show a faster and higher intracellular concentration of melphalan in myeloma cells, as well as lower IC_50_ values than those achieved with free melphalan [25]. The high accumulated concentration of melphalan flufenamide is attributed mainly to its rapid transport into the cells and slow release of the free melphalan out of cells [26].

Melphalan flufenamide is administered intravenously, and it has shown some common adverse effects, such as fatigue, nausea, diarrhea, pyrexia, and respiratory tract infection [24]. It was developed by Oncopeptides AB (Southborough, MA, USA) and approved by the FDA on 26 February 2021 [28].

### 3.3. Voclosporin (Lupkynis^TM^)

**Figure 6:**
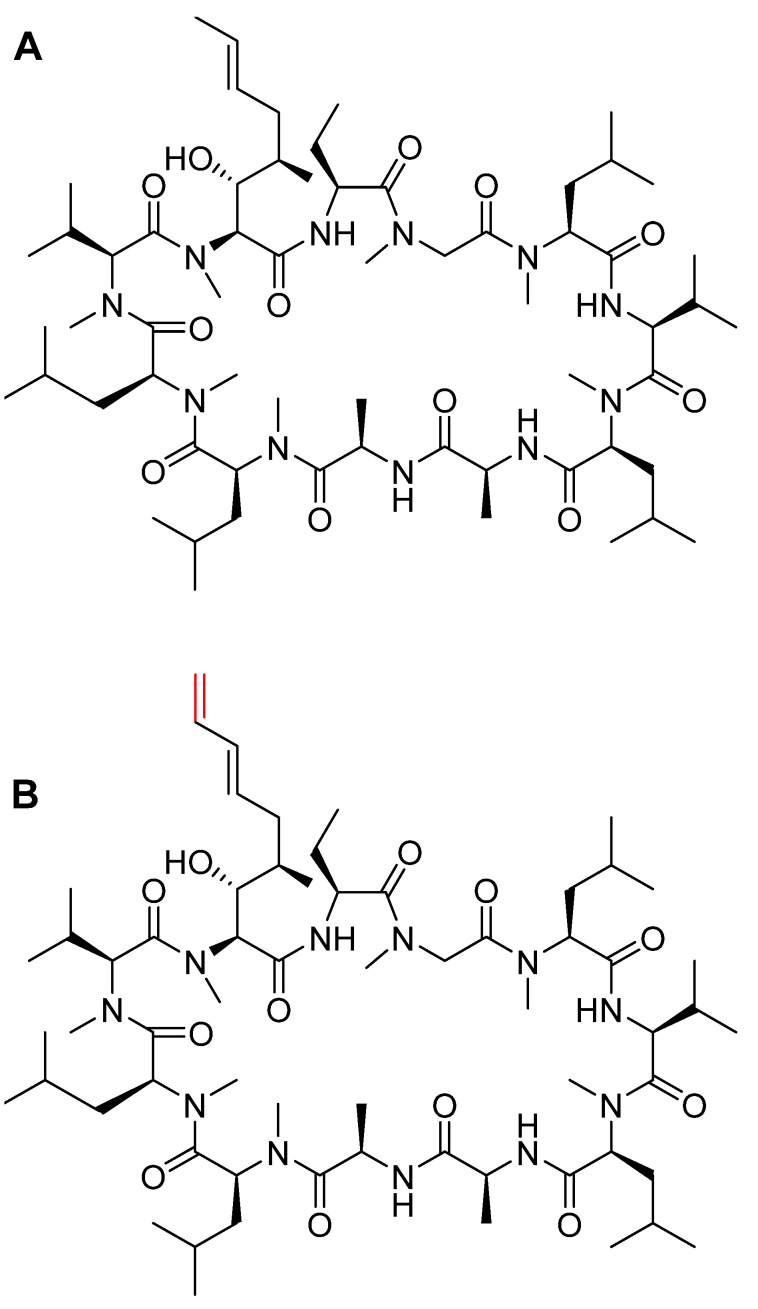
Chemical structures of: **A**. cyclosporin A (CSA). **B**. voclosporin (Lupkynis™). Differences from CSA are shown in red.

Comprising 11 amino acid residues in a cyclic homodetic structure, voclosporin is a novel calcineurin inhibitor analog of cyclosporin A (CSA). Voclosporin contains the same *N-*methyl amino acids as the parent cyclosporin and, in addition, it has an extra double bond in the side chain of the Thr residue (in red) (Figure 6). This modified side chain plays an important role in increasing its potency [29] and also the clearance rate of its metabolites with respect to CSA [30].

It is considered superior in its therapeutic class, and unlike common calcineurin inhibitors, voclosporin comprises a consistent pharmacokinetic profile, thereby removing the need for drug monitoring. In addition, it has a favorable effect on glucose and lipid concentrations [31]. About 99% of the drug is metabolized in the liver by Cytochrome P450 (CYP) 3A4/5 enzyme [32].

It is prescribed for the treatment of lupus nephritis in adults [31]. Like CSA, voclosporin inhibits T-cell-mediated immune response, hence attenuating the inflammatory process and stabilizing the actin cytoskeleton in kidney podocytes, thereby leading to a reduction in proteinuria [33,34].

Voclosporin is administered orally. As it may cause nephrotoxicity, hypertension, neurotoxicity, hyperkalemia, or QT prolongation, various functions of the patient on this drug should be monitored. It is also accompanied by various adverse effects, including decreased glomerular filtration rate, hypertension, diarrhea, headache, anemia, cough, urinary tract infection, upper abdominal pain, dyspepsia, alopecia, renal impairment, abdominal pain, mouth ulceration, fatigue, tremor, acute kidney injury, and decreased appetite [33]. It was developed by Aurinia Pharmaceuticals (Rockville, MD, USA) and approved by the FDA on 22 January 2021 [35].

### 3.4. Pegcetacoplan (Empaveli^TM^)

**Figure 7:**
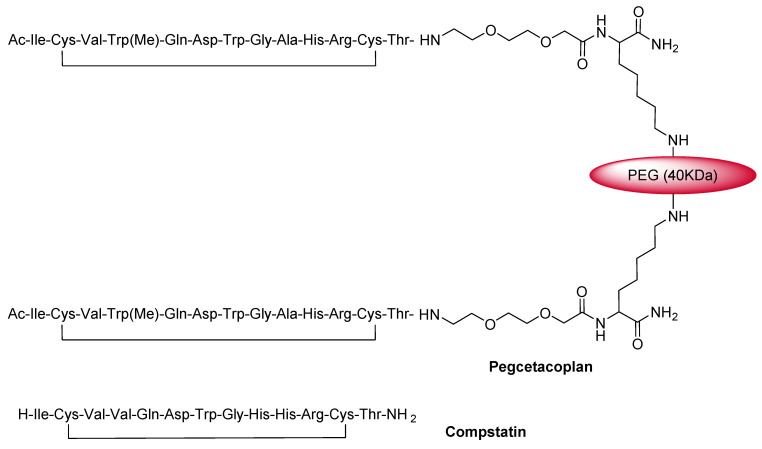
Chemical structures of pegcetacoplan (Empaveli™) and compstatin.

Pegcetacoplan is a C3 inhibitor that is formed by two copies of a tridecapeptide that are covalently conjugated to a linear polyethyleneglycol (PEG) molecule through a Lys linker to enhance its half-life [36] (Figure 7).

Pegcetacoplan is an analog of a cyclic peptide called compstatin (Figure 7). Thus, following the same mode of action, it binds to the complement protein C3 and its activation fragment C3b and regulates the cleavage of C3, as well as the generation of the downstream effectors of complement activation [37].

Pegcetacoplan is the first approved C3-targeted treatment for paroxysmal nocturnal hemoglobinuria (PNH) in adults [37]. PNH is a hemolytic disease caused by a somatic mutation in bone marrow stem cells and in which blood cells lack protective proteins on their surfaces [38]. Pegcetacoplan is also prescribed for patients switching from C5 inhibitor therapy (eculizumab and ravulizumab) [37]. In contrast to C5 inhibitors (eculizumab and ravulizumab), which work downstream of C3, pegcetacoplan targets C3 and acts upstream in the complement cascade, and it shows a broader complement inhibition effect and greater hematological benefit [36]. Furthermore, patients treated with eculizumab can develop clinical manifestations of PNH, and about 72% of patients remain anemic [36]. Hence patients are directed to pegcetacoplan treatment [38].

Developed by Apellis Pharmaceuticals (Waltham, MD, USA), pegcetacoplan was approved by the FDA on 14 May 2021 [39]. Apellis Pharmaceuticals (Waltham, MD, USA) and the University of Pennsylvania (Philadelphia, PA, USA) had an agreement through which the former was granted a worldwide license to develop and commercialize pegcetacoplan in all indications except ophthalmic ones.

It is administered subcutaneously and has some adverse effects, such as infections, diarrhea, abdominal pain, respiratory tract infection, viral infection, and fatigue [37].

### 3.5. Dasiglucagon (Zegalogue^TM^)

**Figure 8:**
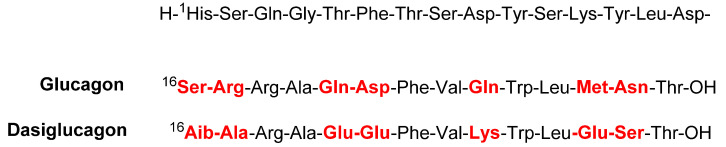
Amino acid sequence of dasiglucagon (Zegalogue^®^) vs. native glucagon.

Dasiglucagon is a glucagon-like hormone peptide with 29 residues. Its structure resembles that of natural glucagon produced by the pancreas. The *N-*terminal sequence (15 amino acids) is common, but 7 amino acids (16, 17, 20, 21, 24, 27, and 28) are substituted by others (red) (Figure 8) to avoid peptide aggregation and enhance solubility and stability [40,41].

Dasiglucagon is used to treat severe hypoglycemia in diabetic patients aged over six years. Hypoglycemia is a condition in which glucose levels in the bloodstream are lower than normal. Dasiglucagon is a glucagon-receptor agonist in hepatocytes. Once bound to the receptors, it activates the breakdown of glycogen and the release of glucose into the bloodstream. Its clinical effect depends on the availability of hepatic glycogen stores [40,42].

Native glucagon is unstable in aqueous solutions. It is provided in powdered form and needs to be dissolved directly before injection. In contrast, due to its solution stability, dasiglucagon is injected subcutaneously either by single-dose injector or by pre-filled syringe, which saves critical time in emergencies [42]. However, this drug has some adverse effects, including nausea, vomiting, headache, and injection site pain in adults and pediatric patients, in addition to diarrhea in the former [41]. The novel dasiglucagon was first developed in 2017 by the Glostrup group and Beta Bionics laboratory [43]. A year earlier, a collaboration agreement was started between Zealand Pharma (Durham, NC, USA) and Beta Bionics laboratory (Concord, MA, USA) to develop an artificial bihormonal pancreas system for the treatment of type 1 diabetes mellitus (T1DM). The pancreas platform technology was founded by Boston University (Boston, MA, USA) and was then consolidated into a wearable pocket-sized device, iLet^(TM)^ [42]. The collaboration was further boosted in 2017, and dasiglucagon was finally approved by the FDA on 22 March 2021 [44].

### 3.6. Piflufolastat F 18 (Pylarify^TM^)

**Figure 9:**
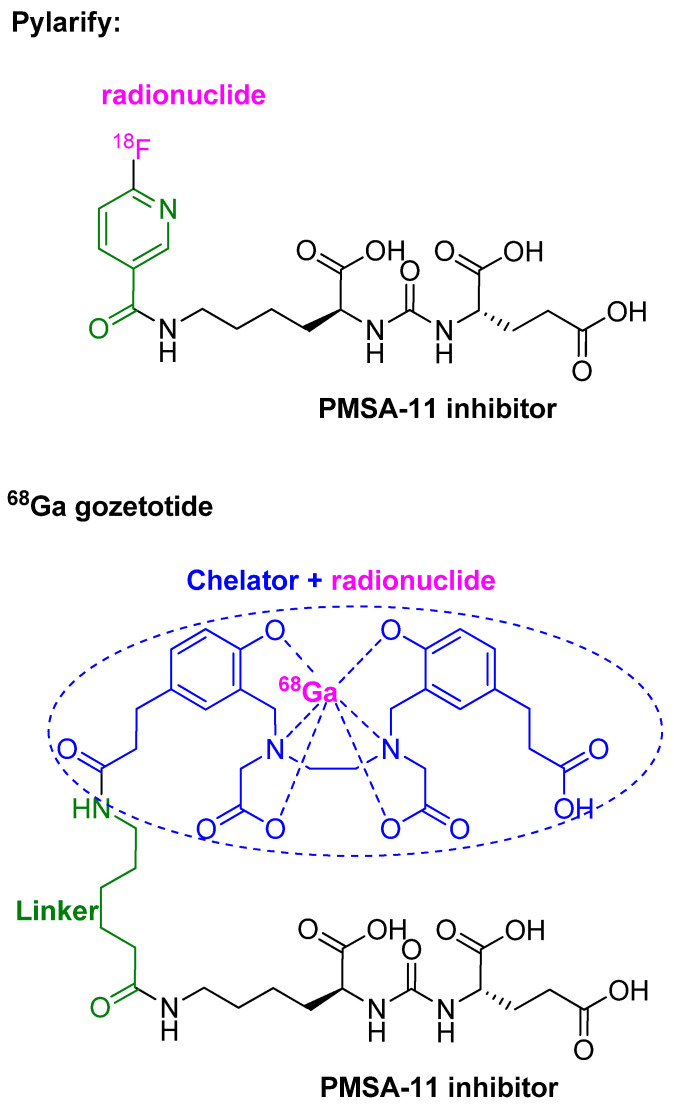
Chemical structure of piflufolastat F18 (Pylarify^TM^) vs. ^68^Ga gozetotide.

Piflufolastat F 18 is a diagnostic peptidomimetic agent labeled with ^18^F radionuclide. It is composed of a urea-based HO-Glu-NH-CO-NH-Lys-OH (black), which is a PMSA-11 inhibitor, attached to 6-[^18^F]fluoro-pyridine-3-carbonyl through the ε-NH_2_ of the Lys residue (Figure 9) [45].

Piflufolastat F18 injection is indicated for positron emission tomography (PET) of prostate-specific membrane antigen (PSMA)-positive lesions in men with prostate cancer with suspected metastasis who are candidates for initial definitive therapy, or with suspected recurrence based on elevated serum prostate-specific antigen (PSA) levels [45,46]. It is the second generation of the PSMA-based PET drug for the same diagnostic purpose after ^68^Ga gozetotide (Figure 9), which was approved by the FDA in 2020 [47,48].

The PMSA-11 antigen moiety drives the drug to PSMA, which is a membrane glycoprotein expressed in several body tissues, especially in the prostate, and overexpressed in tumor cells [48].

Piflufolastat F18 is administered intravenously and has a half-life of 1010 min, thus effectively enabling the imaging and diagnosis of the lesions in 60 min after administration [48]. However, it can cause headaches, dysgeusia, and fatigue [45]. It was developed by Progenics Pharmaceuticals, Inc. (New York, NY, USA) and approved by the FDA on 27 May 2021 [46].

### 3.7. Difelikefalin (Korsuva^TM^)

**Figure 10:**
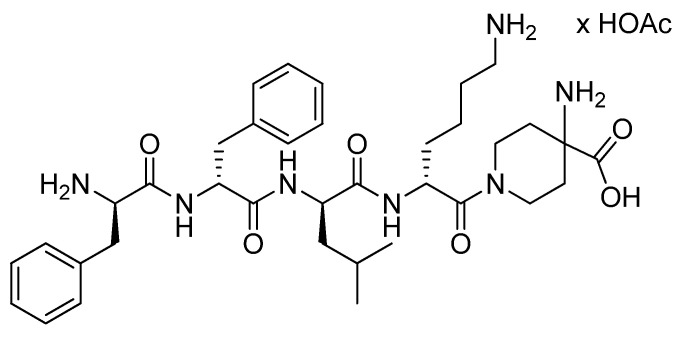
Chemical structure of Korsuva^TM^ (difelikefalin).

Difelikefalin (Korsuva^TM^) is the acetate salt of a tetrapeptide with all D-amino acid residues, FFLK, linked through its C-terminus to 4-aminopiperidine-4-carboxylic acid moiety through the latter’s secondary amine group (Figure 10) [49,50,51].

It is indicated for the treatment of moderate-to-severe pruritus associated with chronic kidney disease (CKD-aP) in adults undergoing hemodialysis [49,52]. Difelikefalin is an agonist that activates the Kappa opioid receptor in nerve cells and reduces the severity of pruritus through an unknown mechanism [50].

Difelikefalin is administered intravenously [50]. It has some adverse effects, such as diarrhea, dizziness, nausea, gait disturbances, including falls, hyperkalemia, headache, somnolence, and mental status change [49]. Its development started in 2011 and was completed by agreements between Cara Therapeutics (Stamford, CT, USA) and other laboratories, and it was registered as a patent, which extends until 2027 [50]. It received its first FDA approval on 23 August 2021 [52].

### 3.8. Odevixibat (Bylvay^TM^)

**Figure 11:**
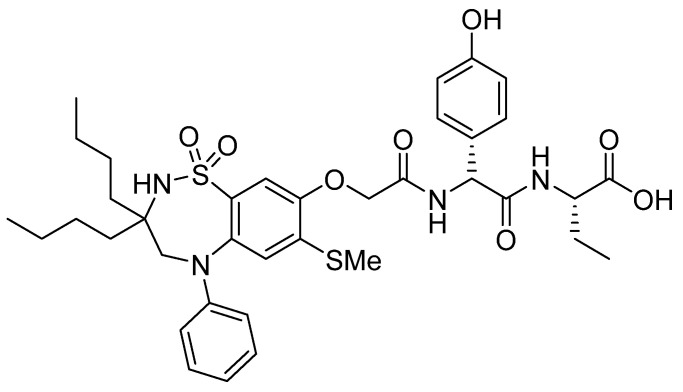
Chemical structure of odevixibat (Bylvay^TM^).

Odevixibat is a small molecule whose structure contains a dipeptide consisting of two non-proteinogenic amino acids, namely D-4-hydroxyphenylglycine and L-ethylglycine. The former, through its N-terminal, is attached to a moiety containing a dioxidothiadiazepin derivative (Figure 11) [53].

It is prescribed for the treatment of pruritus in patients over three months of age with progressive familial intrahepatic cholestasis (PFIC) [53,54,55]. PFIC is a disorder that results from the accumulation of bile acids (gall) in blood serum due to a defect in biliary epithelial transporters (BSEP) between hepatocytes and canaliculus [56]. Odevixibat selectively targets and inhibits the ileal bile acid transporter (IBAT) in the distal ileum (the part between the small and large intestine), lowering the reuptake of bile acids from the intestines to blood serum and clearing the colon of them, thereby reducing their level in blood serum [55,57]. It has reduced efficiency in PFIC patients with mutations that cause non-functional or complete loss of BSEP pumps [53]

It is administered orally and may cause liver test abnormalities, diarrhea, abdominal pain, vomiting, and fat-soluble vitamin deficiency [53]. It was developed by Albireo Pharma Inc. (Boston, MA, USA) and was first approved in the EU (July 2021) for patients aged over six months [55]. Soon after, on 20 July 2021, it was authorized by the FDA for the treatment of patients aged over three months [54].

## 4. Peptides in Antibody-Drug Conjugates (ADCs)

ADCs comprise three main components: antibody, payload or cytotoxin, and linker. Given their high potency and ease with which they can be functionalized for appropriate linking to the rest of the ADC, peptides can be used as payloads. Furthermore, the recognition of peptides by proteases overexpressed by tumors makes them highly suitable components of reversible linkers. In 2021, the two ADCs approved have linkers based on two distinct dipeptides, namely Val-Cit and Val-Ala, and one of them has a peptide as a payload.

### 4.1. Tisotumab Vedotin-Tftv (TIVDAK^TM^)

**Figure 12:**
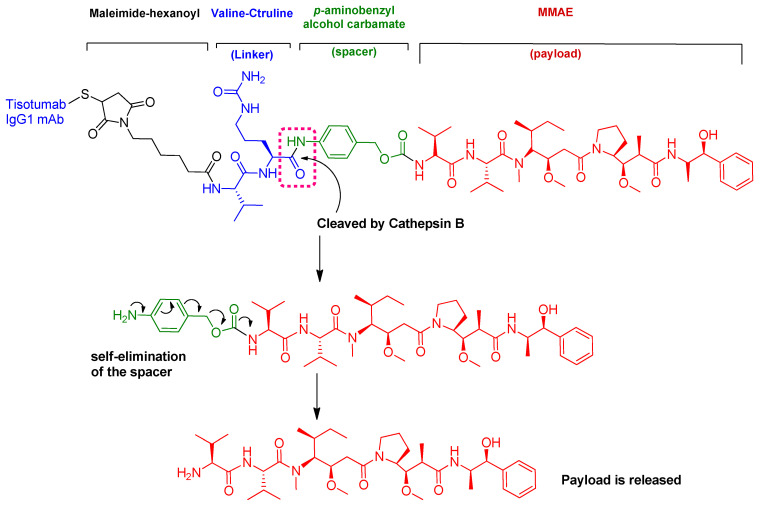
Chemical structure of tisotumab vedotin-tftv (TIVDAK™). Mechanism of payload release with Val-Cit linker and p-aminobenzyl carbamate.

Tisotumab vedotin is an ADC (Figure 12) in which both the linker and payload are peptides [58].

The antibody involved is a human monoclonal antibody that targets tissue factor (TF-011), also known as thromboplastin, factor III, or CD142. The antibody is conjugated to monomethyl auristatin E (MMAE) via a protease-cleavable linker (Val-Cit). MMAE is a synthetic cytotoxin that was engineered to target TF-011-expressing tumor cells without disrupting their role in coagulation [59]. Interestingly, the same payload, as well as the linker, were also used in the previously approved ADCs PADCEV^TM^ and POLIVY^TM^ [12].

Several mechanisms of action are considered for tisotumab vedotin-tftv: (i) MMAE-mediated cell cycle arrest and apoptosis of both tumor and bystander cells and induction of immune cell death; (ii) Fc receptor-mediated effector, including antibody-dependent cellular toxicity (ADCC) and antibody-dependent cellular phagocytosis (ADCP); and (iii) antigen-binding fragment-mediated inhibition of protease-activated receptor-2 (PAR-2)-dependent signaling [59].

Tisotumab vedotin is prescribed for the treatment of recurrent or metastatic cervical cancer with disease progression during or after chemotherapy. It was co-developed by Seagen Inc. (Bothell, WA, USA) and Genmab A/S (Copenhagen, Denmark) and approved by the FDA on 20 September 2021 [60]. It is administered intravenously. Common adverse effects are decreased hemoglobin, fatigue, decreased lymphocytes, nausea, peripheral neuropathy, alopecia, epistaxis, adverse conjunctival reactions, hemorrhage, decreased leukocytes, increased creatinine, dry eye, increased prothrombin international normalized ratio, prolonged activated partial thromboplastin time, diarrhea, and rash [61].

### 4.2. Loncastuximab Tesirine-Lpyl (Zynlonta^TM^)

**Figure 13:**
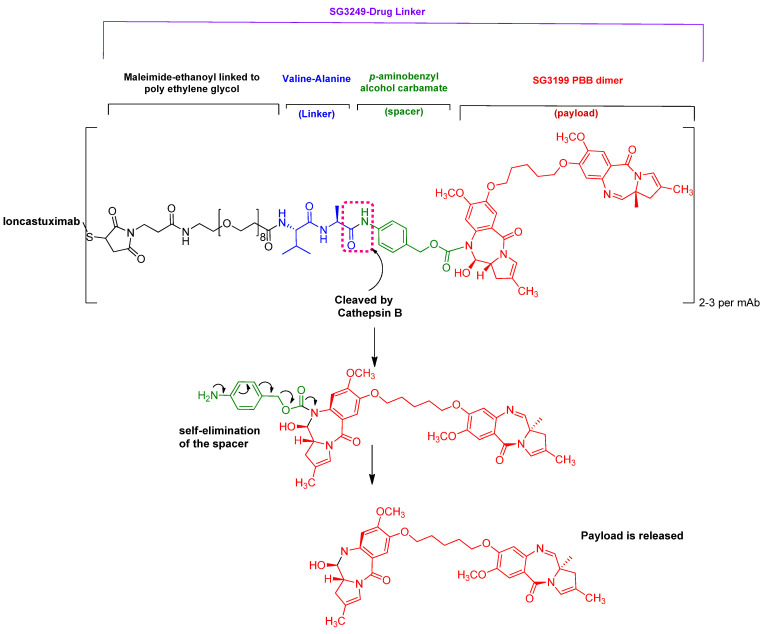
Structure of loncastuximab tesirine-lpyl (Zynlonta^TM^) and mechanism of payload release.

It is an ADC composed of an antibody for targeting CD19 protein conjugated to the payload SG3199 through a maleimido-polyethyleneglycol-dipeptide linker and a spacer (Figure 13) [62].

Zynlonta is indicated for the treatment of adults with relapsed or refractory large B-cell lymphoma [62,63]. Loncastuximab targets CD19 transmembrane protein, which is expressed on the surface of cells of B-lineage origin. This binding causes cell uptake of the conjugate [62].

In the scope of this review, the feature of interest is the dipeptide linker Val-Ala, whose mechanism of payload release is similar to that of the Val-Cit linker. Once inside the tumor cell, the cathepsin B enzyme cleaves the amide bond between the linker and the spacer, then self-elimination of the spacer takes place, freeing the payload inside the cell [64]. The payload SG3199 is an alkylating agent that targets DNA and causes crosslinking, thus bringing about cell death [62].

Zynlonta is administered intravenously, and it has some adverse effects, such as thrombocytopenia, increased gamma-glutamyltransferase, neutropenia, anemia, hyperglycemia, elevated transaminases, fatigue, hypoalbuminemia, rash, edema, nausea, and musculoskeletal pain [62]. It was developed by ADC Therapeutics SA (Murray Hill, NJ, USA) and was granted accelerated approval by the FDA on 23 April 2021 [63].

## 5. Conclusions and Perspectives

A total of eight peptides, two oligonucleotides, and two ADCs containing peptides out of fifty drugs have been approved by the FDA during 2021. The continuous approval of TIDES reflects the importance of this class of drugs, which offer high specificity, efficacy, and also tolerable safety profiles. The consolidation of TIDES has come about for two reasons: the continuous advancement in the synthetic methodologies for large and difficult peptides and, very recently, oligonucleotides, and the development of strategies that allow for more stable peptides and oligonucleotides.

As pointed out in our previous article about the predicted approval of inclisiran [2], we are also expecting further approvals for the TIDES family in the coming years. Regarding inclisiran, the question is now whether it or other next-generation oligonucleotides will substitute statins in the future.

Finally, we believe that research and industrial settings should engage in more effective collaboration to ensure compliance with legislation regarding green synthetic methodologies, thereby allowing them to be ready for the new era of pharmaceutical production.

## Figures and Tables

**Table 1:** Summary of 2021 FDA-approved TIDES.

#	Active Ingredient (Trade Name)	Indication	Therapeutic Target	Administration Route
Oligonucleotides				
1	Inclisiran (Leqvio^TM^)	Treatment of hypercholesterolemia	PCSK mRNA	Subcutaneously
2	Casimersen (Amondys 45)	Duchenne muscular dystrophy (DMD) amenable for exon 45 skipping	Exon 45	Intravenously
Peptides				
3	Vosoritide (Voxzogo™)	Achondroplasia	Natriuretic peptide receptor B (NPR-B)	Subcutaneously
4	Melphalan flufenamide (Pepaxto^TM^)	Treatment of multiple myeloma (MM) and amyloid light-chain amyloidosis	Exerts anti-tumor activity through crosslinking of DNA	Intravenously
5	Voclosporin (Lupkynis™)	Treatment of lupus nephritis	T-cells	Orally
6	Pegcetacoplan (Empaveli™)	Treatment of paroxysmal nocturnal hemoglobinuria (PNH) in adult patients	Complement protein C3 and its activation C3b	Subcutaneously
7	Dasiglucagon (Zegalogue^TM^)	Hypoglycemia in diabetes patients aged over 6 years	Glucagon-receptor	Subcutaneously
8	Piflufolastat-F^18^ (Pylarify^TM^)	Positron emission tomography (PET) of prostate-specific membrane antigen (PSMA)-positive lesions in men with prostate cancer	PSMA	Intravenously
9	Difelikefalin (Korsuva^TM^)	Pruritus associated with chronic kidney disease (CKD-aP) in adults undergoing hemodialysis (HD)	Kappa opioid receptor	Intravenously
10	Odevixibat (Bylvay^TM^)	Pruritus in patients aged over 3 months with progressive familial intrahepatic cholestasis (PFIC)	Ileal bile acid transporter (IBAT)	Orally
Peptides in ADCs				
11	Tisotumab vedotin-tftv (TIVDAK™) (MMAE as cytotoxic and Valine-Citrulline as linker)	Treatment of recurrent or metastatic cervical cancer with disease progression during or after chemotherapy	Tissue factor (TF-011),	Intravenously
12	Loncastuximab tesirine-lpyl (Zynlonta^TM^) (Valine-Alanine as linker)	Treatment of adults with relapsed or refractory large B-cell lymphoma	CD19	Intravenously

## Data Availability

Not applicable.
